# Management of adult-onset Still's disease: evidence- and consensus-based recommendations by experts

**DOI:** 10.1093/rheumatology/kead461

**Published:** 2023-09-05

**Authors:** Helen L Leavis, Paul L A van Daele, Catharina Mulders-Manders, Renée Michels, Abraham Rutgers, Elizabeth Legger, Marc Bijl, Elisabeth A Hak, Wai-Kwan Lam-Tse, Femke Bonte-Mineur, Peter Fretter, Anna Simon, Pieter van Paassen, Marlies C van der Goes, Marcel Flendrie, Ward Vercoutere, Antoine W T van Lieshout, Arjen Leek, Sebastiaan J Vastert, Sander W Tas

**Affiliations:** Department of Rheumatology and Clinical Immunology, University Medical Center Utrecht, Utrecht, The Netherlands; Department of Immunology, Erasmus Medical Center, Rotterdam, The Netherlands; Department of Internal medicine, Radboud University Medical Center, Nijmegen, The Netherlands; IQVIA, Amsterdam, The Netherlands; Department of Rheumatology and Clinical Immunology, University Medical Center Groningen, Groningen, The Netherlands; Department of Pediatric Rheumatology, University Medical Center Groningen, Groningen, The Netherlands; Department of Rheumatology and Clinical Immunology, Martini Hospital, Groningen, The Netherlands; Department of Rheumatology and Clinical Immunology, Amsterdam University Medical Center, Amsterdam, The Netherlands; Department Rheumatology, Franciscus Hospital, Rotterdam, The Netherlands; Department of Rheumatology and Clinical Immunology, Maasstad Hospital, Rotterdam, The Netherlands; Department of Rheumatology, Treant Hospitals, Emmen/Hoogeveen/Stadskanaal, The Netherlands; Department of Internal medicine, Radboud University Medical Center, Nijmegen, The Netherlands; Department of Nephrology and Clinical Immunology, Maastricht University Medical Center, Maastricht, The Netherlands; Department of Rheumatology, Meander Medical Center, Amersfoort, The Netherlands; Department of Rheumatology, Maartenskliniek, Nijmegen, The Netherlands; Department of Rheumatology, Reumazorg Zuid-West Nederland, Goes-Terneuzen-Oostburg, The Netherlands; Department of Rheumatology, Jeroen Bosch Ziekenhuis, ’s-Hertogenbosch, The Netherlands; Department of Pediatrics, University Medical Center Utrecht, Utrecht, The Netherlands; Department of Pediatrics, University Medical Center Utrecht, Utrecht, The Netherlands; Department of Rheumatology and Clinical Immunology, Amsterdam University Medical Center, Amsterdam, The Netherlands

**Keywords:** Adult-onset Still’s disease, Delphi, IL-1, Still’s disease, treatment, guideline, diagnosis

## Abstract

**Objectives:**

Adult-onset Still’s disease (AOSD) is a rare condition characterized by fevers, rash, and arthralgia/arthritis; most doctors treating AOSD in the Netherlands treat <5 patients per year. Currently, there is no internationally accepted treatment guideline for AOSD. The objectives of this study were to conduct a Delphi panel aimed at reaching consensus about diagnostic and treatment strategies for patients with AOSD and to use the outcomes as a basis for a treatment algorithm.

**Methods:**

The Delphi panel brought together 18 AOSD experts: rheumatologists, internists and paediatricians. The Delphi process consisted of three rounds. In the first two rounds, online lists of questions and statements were completed. In the third round, final statements were discussed during a virtual meeting and a final vote took place. Consensus threshold was set at 80%. Two targeted literature searches were performed identifying the level of evidence of the consensus-based statements.

**Results:**

Consensus was reached on 29 statements, including statements related to diagnosis and diagnostic tests, definition of response and remission, the therapy, the use of methotrexate and tapering of treatment. The panel consented on reduction of the use of glucocorticoids to avoid side effects, and preferred the use of biologics over conventional treatment. The role of IL-1 and IL-6 blocking agents was considered important in the treatment of AOSD.

**Conclusion:**

In this Delphi panel, a high level of consensus was achieved on recommendations for diagnosis and therapy of AOSD that can serve as a basis for a treatment guideline.

Rheumatology key messages

- Corticosteroids are effective in AOSD but should be avoided because of side effects.
- Early initiation of biologics is preferred over conventional treatment such as methotrexate.
- There is an important role for early use of IL-1 and IL-6 blockade in AOSD.

## Introduction

Adult onset Still’s disease (AOSD) is a rare systemic autoinflammatory condition characterized by fever, arthralgia/(poly)arthritis and a salmon-coloured skin rash. Other symptoms include hepatosplenomegaly, lymphadenopathy and neutrophilia [1]. Systemic juvenile idiopathic arthritis (sJIA, Still’s disease) shows many similarities and SJIA and AOSD are increasingly seen as part of the same clinical disease entity. Their aetiology is largely unknown.

As AOSD is primarily a diagnosis of exclusion, a significant diagnostic delay is often present, increasing the chance of complications. To shorten the time until diagnosis, there is a need for a clear diagnostic workflow.

Anti-inflammatory drugs form the cornerstone of treatment of AOSD. Treatment is often based on expert opinion through the lack of international guidelines. Historically, glucocorticoids (GC) are considered the backbone of treatment. MTX is often the first conventional DMARD used to spare GC [2]. More recently, IL-1β and IL-6 were identified as important mediators of inflammation in AOSD. Biologics targeting these pathways are approved by both the FDA and the EMA for the treatment of sJIA and AOSD.

Given the rarity of AOSD, most Dutch physicians who treat these patients treat <5 patients a year, resulting in limited treatment experience. There is no internationally accepted guideline for the treatment of AOSD. The optimal timing of initiation of conventional DMARDs and interleukin-blocking therapy remains unresolved [3]. To fill the remaining gap, we performed a targeted literature review and Delphi-based study and propose a treatment algorithm for AOSD.

## Methods

### Design

The aim of a (modified) Delphi exercise is to reach consensus between experts on a specific topic where insufficient evidence-based proof exist [4] by systematically synthesizing the knowledge and opinions of a large group of individuals with diverse expertise and from different geographic locations [5].

A three-member core group (H.L., P.D. and S.T.), who have all treated over 15 AOSD patients in the previous 15 years, initiated the Delphi panel study. All three core members work in a university hospital. Two core members are internist-clinical immunologists and one is an internist- rheumatologist. They were supported by one paediatric rheumatologist (B.V.) who led the design of treatment guidelines for sJIA [6].

The other Delphi panel members were selected by nomination by the core group and upon advice of colleagues. Regional spread was a criterion. A second paediatrician with expertise in sJIA was involved. The total panel consisted of 17 participants, of whom 10 (59%) work in a university hospital, the others in a community hospital. There were nine adult rheumatologists (53%), six internists (35%) and two paediatric rheumatologists. For further characteristics of the participants, see Supplementary Tables S1 and S2 (available at *Rheumatology* online). All participants gave their opinion during the third and final virtual meeting, although three were not present but responded in parallel using e-mail.

### Background

Before the first Delphi round, relevant literature on (i) evidence regarding MTX in Still’s disease; (ii) a recent overview on IL-1 blockade in Still’s disease [7], and (iii) the Dutch treatment guideline on (s)JIA [8] was identified. During a teleconference all participants were informed on the background and aim of the Delphi panel and the aim to discuss the literature.

### Literature review

A systematic literature review (for search string see Supplementary Data S1–S3, available at *Rheumatology* online) was performed by the core group, using MEDLINE via PubMed and EMBASE, with an end date of 31 January 2020. A summary was shared with the panellists together with the first round Delphi questions. Evidence was scored using the GRADE system [9].

### Consensus development process

The first round consisted of 27 open ended, nominal, and yes or no questions with the subjects of (i) demographics of the participants; (ii) diagnosis of AOSD; (iii) definitions of response and remission in AOSD; and (iv) treatment of AOSD (e.g. optimal timing, reduction of GC use, biologics).

Based on the results, a total of 37 statements were formed, partly inspired by the Italian Delphi panel study [3]. In round 2, aggregated results and the 37 consensus statements were presented for agreement. If a respondent did not agree, they were asked to provide further arguments for their disagreement. A total of 21 of 37 statements reached consensus (between >85% of respondents) after the second round.

All statements not reaching consensus were re-worded for the third round by the core group, using the disagreeing arguments from round 2. An online portal (Dynata) was used for round 1 and 2.

In May 2020 the statements that did not reach consensus during round 2 were discussed during a virtual meeting (Microsoft Teams). The meeting was moderated by J.F., a paediatric rheumatologist with specific expertise in hereditary autoinflammatory syndromes. Using a rotation method three new participants were asked to open the discussion for each statement, after which statements were voted on (raising a virtual hand, via chat function, or verbally). If needed, statements were reworded and/or merged during the virtual meeting. The total adjusted number of statements voted on during round 2 and 3 was 33 (Supplementary Tables S3 and S4, available at *Rheumatology* online). Consensus level was set at 80% agreement [10].

### Ethics

Due to the Delphi design no patient data were used. No ethics approval was obtained for this project, since informed consent was non-applicable.

## Results

**Figure 1.**
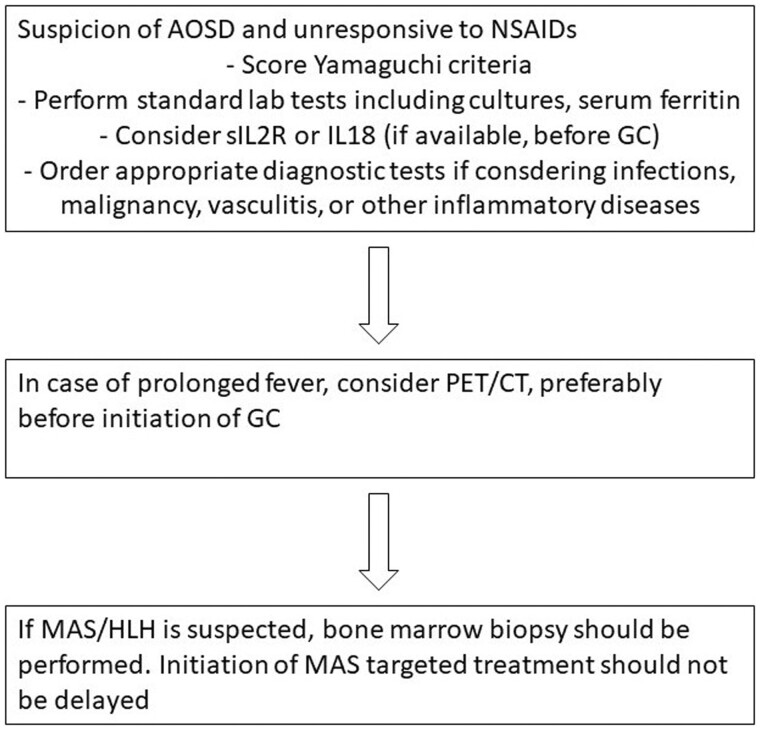
Diagnostic workup of (suspected) AOSD. AOSD: adult onset Still’s disease; GC: glucocorticoids; HLH: haemophagocytic lymphohistiocytosis; MAS: macrophage activation syndrome

**Figure 2.**
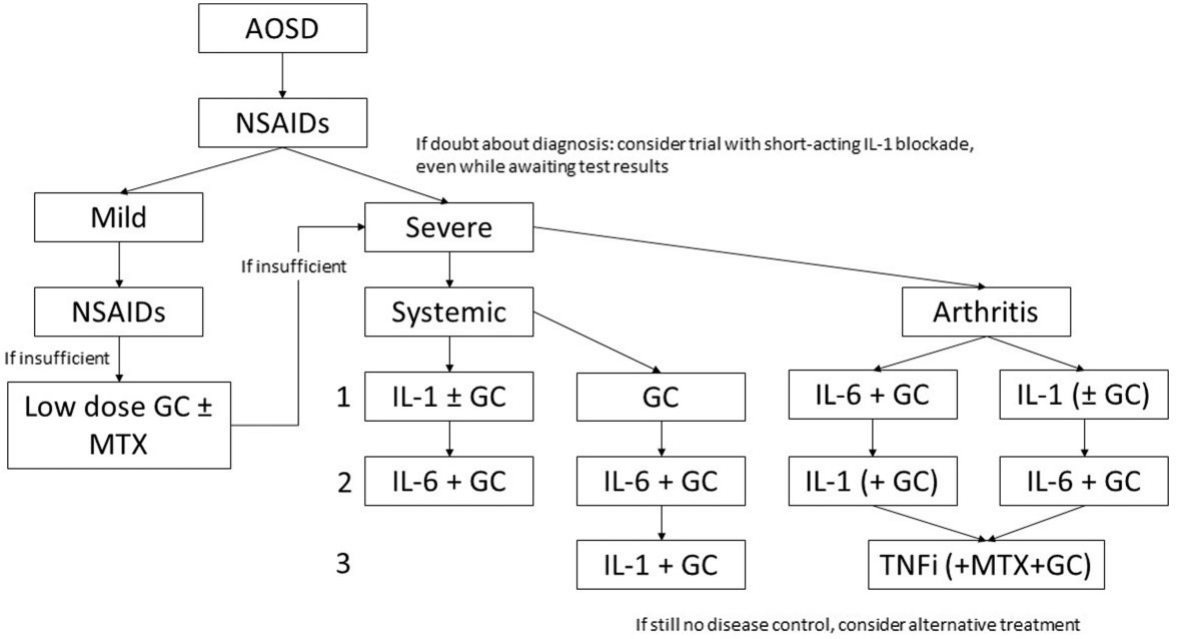
Suggested treatment algorithm for patients with AOSD. Severe disease is defined as hospital admission needed, with organ involvement or life-threatening disease. Systemic symptoms: fever, weight loss, sore throat, skin rash, lymphadenopathy, hepatosplenomegaly. In case of predominant arthritis, IL-6 blockade may be preferred over IL-1 blockade. Low-dose glucocorticoid is ≤15 mg prednisone or equivalent. Double arrows indicate equivalent options and a decision should be made upon the physician’s discretion. GC refers to high dose glucocorticoid, i.e. prednisone 0.5–1.0 mg/kg or equivalent. ‘IL-1’ indicates IL-1 blockade; ‘IL-6’ indicates IL-6 blockade. MTX dosing according to EULAR; IL-1 blockade 100–300 mg subcutaneously or off label intravenously. AOSD: Adult onset Still’s Disease; GC: glucocorticoid; TNFi: TNF inhibitors

At the end of round 3, after rewording and merging, a total of 29 of 33 final statements achieved consensus (Table 1, Supplementary Tables S3 and S4, available at *Rheumatology* online). Based on these statements, we propose diagnostic and treatment algorithms (Figs 1 and 2 and Supplementary Figs S1–S3, available at *Rheumatology* online).

**Table 1.** Main consensus statements used for the formation of a treatment algorithm for AOSD and level of evidence

	Statement	Agreement	Level of evidence	Grade	References
1	High dose glucocorticoids are effective in AOSD, but due to side effects, the aim should be to limit the frequency and duration of treatment	100%	2B	B	[1, 6, 11–13]
2	IL-1 blockade is an effective treatment early in the AOSD disease course, including in glucocorticoid naïve patients	88%	2B	B	[12, 14–17]
3	In case of life-threatening symptoms of AOSD, treatment with a combination of glucocorticoids with a biologic or glucocorticoid monotherapy should be started as soon as possible	88%	2B	C	[2, 12, 13]
4	If bacterial sepsis is unlikely, starting with short-acting IL-1 blockade in case of life-threatening and rapidly progressing AOSD is justified even if not all test results are known yet	100%	1B	B	[15] and expert opinion
5	In case treatment is started with short-acting IL-1 blockade, the diagnostic process is disrupted less than when starting GC (besides a possible decrease in serum cytokine levels)	100%	4	D	Expert opinion
6	IL-6 blockade is a treatment option in glucocorticoid-resistant or glucocorticoid-dependent AOSD	100%	2B	C	[18–30]
7	IL-1 or IL-6 blockade is preferred in AOSD over TNF blockade; however, TNF blockade may be useful in some patients with persistent arthritis (without systemic manifestations) in the course of their disease	100%	5	D	[18, 21–25, 27–36]
8	Adding MTX may be useful for the treatment of arthritis in AOSD, but there is little evidence for treatment in AOSD with systemic features	94%	4	C	[37–47]

AOSD: adult-onset Still’s disease.

### Diagnostic approach

The statements on the approach to a patient with suspected AOSD are mainly based on expert opinion. The panel’s statements and considerations on diagnostics are presented in Table 1, Supplementary Table S3, available at *Rheumatology* online and visualized in Fig. 1. AOSD mainly remains a diagnosis of exclusion and its differential diagnosis is broad and includes infections, non-infectious inflammatory diseases, and malignancies [48]. Kontzias *et al.* have suggested a standard diagnostic approach for AOSD [49]. Scoring of the Yamaguchi criteria [50] is required. If available, measurement of serum soluble IL-2 receptor (sIL-2R) or IL-18 should be considered, preferably before the start of GC. Macrophage activation syndrome (MAS)/haemophagocytic lymphohistiocytosis (HLH) is a major complication of AOSD and present in up to 23% of patients [51].

### Treatment of AOSD

The statements that were used as a basis for the treatment algorithm (Fig. 2, Supplementary Figs S1–S3, available at *Rheumatology* online) are shown in Table 1. Note that for practical purposes AOSD treatment response and disease remission, respectively, were defined by the panel as decrease in serum ferritin, recovery of clinical symptoms, decrease in CRP and/or ESR (consensus 100%); and normalization of serum ferritin, absence of clinical symptoms, normalization of CRP and/or ESR (consensus 94%) [19]. For validity of extrapolation from children to adults two statements on the similarity between sJIA and AOSD were included [3] (Supplementary Table S3, available at *Rheumatology* online):

- The clinical presentation of sJIA is very similar to that of AOSD and therefore these conditions can be considered as expressions of the same disease spectrum [3] (grade C)
- Early use of biologics can improve outcomes in adults with AOSD (grade D)

### Glucocorticoids

With the literature search (See Supplementary Data S1, available at *Rheumatology* online), 132 publications were found, of which only two were relevant: one case series of adolescents with a clinical picture fitting AOSD [11] and one review on juvenile chronic arthritis [13]. Broadening the search (see Supplementary Data S1, available at *Rheumatology* online) yielded 74 results. None specifically examined the effect of systemic GC in AOSD. One observational study (Supplementary Table S5, available at *Rheumatology* online) including 80 patients [12] compared high-dose GC (prednisone 0.8–1.0 mg/kg/day or equivalent) to low-dose GC (0.2–0.3 mg/kg/day). Monotherapy with high-dose GC led to a statistically significant higher rate of remissions after 6 months of treatment (65% *vs* 23%; *P* < 0.01). MAS was seen more often in the low-dose group. A case series including five adolescents, of whom three were treated with prednisone 2 mg/kg, reports rapid response after failure of non-steroidal anti-inflammatory drugs [11] (Supplementary Table S5, available at *Rheumatology* online).

Based on these results, two main statements (Table 1, statement 1 and 3) were developed.

### IL-1 Blockade

Studies on the early/initial use of IL-1 blockade in AOSD have not been performed. As the expert panel considered AOSD and sJIA to be part of the same disease spectrum, evidence from sJIA studies was extrapolated. Six studies were identified [3, 14–17, 52] (Table 2).

**Table 2.** Clinical evidence regarding treatment with IL-1 and IL-6 blockade in AOSD

Author	Drug	Design	Number of patients	Reference
IL-1 blockade				
Colafrancesco *et al.*	ANA, RILO	Qualitative	N/A	[3]
Jamilloux *et al.*	ANA	Review	N/A	[14]
Vastert *et al.*/Ter Haar *et al.*	ANA	Prospective	20	[15, 16]
Junge *et al.*	ANA, CANA, RILO	Review	N/A	[17]
Feist *et al.*	ANA, CANA, RILO	Review	N/A	[52]
IL-6 blockade				
Vercruysse *et al.*	TCZ	Retrospective multicentre	27	[19]
Kaneko *et al.*	TCZ	RCT	27	[20]
Li *et al.*	TCZ	Prospective	8	[21]
Ma *et al.*	TCZ	Meta-analysis	147	[22]
Song *et al.*	TCZ	Retrospective multicentre	22	[23]
Bannai *et al.*	TCZ	Case Series	7	[24]
Ortiz *et al.*	TCZ	Retrospective open label	34	[25]
Cipriani *et al.*	TCZ	Open label prospective	11	[26]
Suematsu *et al.*	TCZ	Retrospective	11	[27]
Puechal *et al.*	TCZ	Prospective	14	[28]
		Multicentre		
Elkayam *et al.*	TCZ	Retrospective	15	[29]
Nishina *et al.*	TCZ	Retrospective	40	[30]

ANA: anakinra; AOSD: adult-onset Still’s disease; CANA: canakinumab; N/A: not available; RILO: rilonacept; TCZ: tocilizumab.

An extensive literature review on efficacy and safety of IL-1 blocking therapy in AOSD is available [3]. Median effectiveness is 83.3% (range 50–100%) with median disease remission rate 70% (range 22.2–100%). Median treatment failure rate was 16.7%. In an international multicentre series 22% of 46 patients received recombinant IL-1 receptor antagonist (eIL-1RA) and 80% of these patients attained complete response without escalation of therapy [53]. In a cohort of 20 sJIA patients treated with rIL-1RA, 75% achieved minimal active or inactive disease after 3 months; and this response was sustained in 13/20 patients [15]. After 1 year without medication, 52% of these still had inactive disease [16], and after 5 years 96% had inactive disease and 75% inactive disease without medication.

Based on these results and personal clinical experience of the core group, three main statements on IL-1 blockade were developed (Table 1, statement 2, 4 and 5). Based on personal experience, a statement on reduced interference with the diagnostic trajectory of IL-1 inhibitors compared with GCs was included (Table 1, statement 5).

### IL-6 Blockade

Studies on the effects of IL-6 inhibition are summarized in Table 2. All studies report on the use of tocilizumab (TCZ). TCZ has mainly been studied in refractory AOSD with arthritis. A Japanese trial randomized 27 AOSD patients between biweekly TCZ or placebo [20] for 12 weeks, followed by a 40 week open label study. GC dosages and systemic scores decreased significantly in the TCZ group. In over 160 reported AOSD patients treated with varying dosages of TCZ [19–27], overall clinical response ranged from 64.3% to 100%.

One main statement on the early use of IL-6 blockade was developed (Table 1, statement 6).

### MTX

Many recommendations base their advice on MTX on a retrospective cohort of 26 patients [37]. The evaluation of efficacy of MTX in AOSD results from one prospective multicentre cohort study [38], retrospective [37, 39–43] and monocentre studies [44, 45] and several case series [46, 54], predominantly in patients with polyarticular involvement. The reported efficacy is ∼60–83%. In a randomized trial [45] there was no significant improvement compared with placebo. In an open randomized controlled trial (RCT) comparing anakinra to conventional DMARDS, the number of patients on MTX was too small to draw conclusions [44] (Table 3).

**Table 3.** Clinical evidence regarding treatment with methotrexate in AOSD

Author	Drug	Design	No. of patients	Reference
Fautrel *et al.*	MTX	Retrospective, uncontrolled	26	[37]
Kalyonku *et al.*	MTX	Prospective multicentre	202	[38]
Pay *et al.*	MTX	Retrospective multicentre	61	[39]
Iliou *et al.*	MTX	Retrospective, uncontrolled	11	[40]
Franchini *et al.*	MTX	Retrospective monocentre	27 (five combination of DMARDs)	[41]
Fuji *et al.*	MTX	Retrospective monocentre	13	[42]
Singh *et al.*	MTX	Retrospective	10	[43]
Nordström *et al.*	MTX	RCT	6	[44]
Woo *et al.*	MTX	RCT	45	[45]
Aydintug *et al.*	MTX	Case series	6	[46]
Manger *et al.*	MTX	Review	71	[47]
Wang *et al.*	MTX	Prospective	28	[54]

AOSD: adult-onset Still’s disease.

The reported complication rates (e.g. erosive bone disease) are relatively high despite the use of MTX in cohort studies.

One main statement on the early use of MTX in AOSD was developed (Table 1, statement 8).

### TNF-blockade

Data on TNF-blockade in AOSD is limited (Table 4). No RCTs are present. Its effect is mainly reported in patients with predominantly arthritis who lack systemic symptoms [27, 31–33, 55]. In the presence of systemic symptoms, most patients only achieve partial response. A few patients with refractory disease (including severe liver diseases) responded to TNF-blockade [34, 35, 56, 57].

**Table 4.** Clinical evidence regarding treatment with TNF blockade in AOSD

Authors	Drug	Design	No. of patients	Reference
Suematsu *et al.*	Infliximab, etanercept	Retrospective multicentre	13	[27]
Kraetsch *et al.*	Infliximab	Prospective pilot	6	[31]
Fautrel *et al.*	Infliximab, etanercept	Restrospective multicentre	20	[32]
Dilhuydy *et al.*	Infliximab	Case report	1	[33]
Zhou *et al.*	Infliximab, etanercept, adalimumab	Retrospective multicentre	293	[34]
Babacan *et al.*	Infliximab	Case report	1	[35]
Maeshima *et al.*	Etanercept	Case report	1	[55]
Naniwa *et al.*	Etanercept	Case report	1	[56]
Singh *et al.*	Infliximab	Case report	1	[57]

AOSD: adult-onset Still’s disease.

One main statement was developed on the use of TNF-blockade in AOSD (Table 1, statement 7).

## Discussion

The aim of this study was to reach consensus on diagnosis and treatment of AOSD with the use of a Delphi process. The 29 out of 33 statements that reached consensus between >80% of 17 panelists form the basis for a treatment guideline to be used in clinical practice (Figs 1 and 2 and Supplementary Figs S1–S3, available at *Rheumatology* online).

### Recommendations for the diagnosis of AOSD

When AOSD is considered in the differential diagnosis, standard lab tests, blood and urine cultures, and serum ferritin should be performed. Scoring the Yamaguchi criteria [50] is required (Fig. 1). Individual clinical features determine whether a patient should be tested for alternative diagnoses (infections, non-infectious inflammatory diseases, malignancy) [48, 49]. Measurement of soluble IL-2 receptor or IL-18 should be considered to support the diagnosis of AOSD, preferably before initiation of GC. Timely start of treatment is important to avoid damage or a more chronic disease course. In case of prolonged fever or inflammation, PET/CT should be considered before starting GC. Upon suspicion of MAS, bone marrow examination should be performed, but this should not delay MAS-targeted treatment, as this is a potentially life-threatening complication. If doubt remains about the diagnosis, a trial with short-acting IL-1 blockade should be considered while waiting for test results (Fig. 1).

### Recommendations for the treatment of AOSD

The consent recommendations for treatment of AOSD are the following (Fig. 2):

- NSAIDS can be given in any patient suspected of AOSD.
- The goal remains to limit the use of GC in AOSD, mainly because of the side effects. IL-1 blockade interferes less during the diagnostic trajectory and can be used for a therapeutic trial.
- Mild disease, unresponsive to NSAIDs: short course of GC with or without MTX (dosing and administration see [58]). If after 4–6 weeks GC exceeds 7.5 mg per day, step up to recommendations for more severe disease.
- Severe systemic disease: either monotherapy IL-1 blockade, or GC and IL-1 blockade, or monotherapy GC. Step-up to IL-1 blockade should be considered in insufficient response on GC monotherapy or upon GC dependence.
- Severe arthritis predominant: MTX and GC combination therapy, GC and IL-6 blockade combination therapy; IL-1 blockade and GC combination therapy; or IL-1 blockade monotherapy. IL-6 blockade may be preferred to IL-1 blockade in arthritis-dominant patients, but comparative studies are lacking.
- Severe disease, unresponsive to initial treatment: switch to IL-1 blockade or IL-6 blockade, depending on the initial choice.
- TNF blockade may be used in arthritis-predominant AOSD patients without systemic symptoms and upon suboptimal response to both IL-1 and IL-6 blockade.

The initial dose of short-acting IL-1 blockade (i.e. anakinra) should depend on disease severity and could range from 100 to 400 mg subcutaneously per day [8]. Dosing over 100 mg is off-label but has proven to be safe in sepsis trials [59] and multiple COVID-19 trials (reviewed in [60]). In children, doses up to 8 mg/kg have been proven safe [15]. In cases with insufficient response within 2 days of monotherapy, the dose can be escalated. For rapid progressive or life-threatening disease, off-label intravenous dosing of anakinra, 200–400 mg per day, in combination with GC or monotherapy GC can be considered. Pulsatile methylprednisolone intravenously is preferred over long-term high-dose oral GC. Intravenous immunoglobulin treatment is optional. If a patient deteriorates despite this in absence of an alternative diagnosis, case reports and case series describe alternative and rescue therapies for AOSD and sJIA, such as cyclophosphamide [61–64], anti-thymocyte globulin [65], intravenous immunoglobulin (IvIg) [66] or etoposide [67, 68]. The human anti-IFN-γ antibody emapalumab showed beneficial effect in 14 patients with sJIA/AOSD-associated macrophage activation syndrome [69]. A new recombinant human IL-18 binding protein (tadekinig alfa) needs to be further evaluated, but showed favourable safety and modest efficacy in a 12-week phase 2 trial [70].

In case of disease remission, GC should be tapered to ≤7.5 mg prednisolone or equivalent daily. If disease remission is obtained for ≥3 months while on short-acting IL-1 blockade 100 mg s.c. daily, the dose can be tapered to every other day for 4–6 weeks and stopped upon persisting remission. If a patient is in a 3-month remission on long-acting IL-1 blockade (canakinumab) monotherapy, the dose of canakinumab can be tapered by increasing the interval of administration.

The current study is the first AOSD consensus study in the Netherlands. In the absence of a strongly evidence-based approach for the diagnosis and treatment of AOSD, this work provides an important milestone in optimizing treatment for patients with AOSD. The Delphi panel was elaborate, the participation rate of clinicians with expertise on AOSD was high, and the panel included an adequate representation of AOSD experts in the Netherlands. Because sJIA and AOSD were considered a spectrum by the panel, study results were to some extent extrapolated from children to adults. Compared with other recent treatment recommendations [71, 72], we advocate the early use of biologics over conventional treatments such as MTX, especially in patients with severe systemic disease. It should be noted that many of the consensus-based statements are based on low level evidence, because of the rarity of AOSD and the scarcity of clinical trials. Comparative studies are needed to validate the recommendations made here. A potential limitation to our Delphi process is that the third and final meeting was held virtually, and because of this anonymity was not preserved, although the Delphi panel method is intended specifically to counteract the effects of any psychological pressure that can influence opinions in a face-to-face, small group discussion environment. The systematic method or rotation for input was used to circumvent psychological pressure.

In conclusion, this paper presents the results of a modified Delphi process that resulted in 29 consensus-based statements on the diagnosis and treatment of AOSD, which form the basis for both a diagnostic and a therapeutic algorithm.

## Supplementary Material

kead461_Supplementary_Data

## Data Availability

Data are available from the corresponding author upon request.
