# Supplementary material for: Management of adult-onset Still's disease: evidence- and consensus-based recommendations by experts
Source: Rheumatology (Oxford). 2023 Sep 5;63(6):1656–63. doi: 10.1093/rheumatology/kead461 (PMC11147545; doi:10.1093/rheumatology/kead461)
Supplement: kead461_Supplementary_Data [file kead461_supplementary_data.zip › kead461_Supplementary_Data/rhe-23-0590-File004.docx]

**Supplementary material**

| **Supplementary table S1. How many AOSD patients are treated in your hospital each year (new patients and follow-up together)?** | | |
| --- | --- | --- |
| **Average (90% CI)** | | **14;10,85-17,62** |
| Average amongst academic hospitals (90% CI) | | 18;13,48-23,19 |
| Average amongst general hospitals (90% CI) | | 10; 6,50-12,75 |
| - 0 to 10 | | 2 |
| - 5 to 10 | | 3 |
| - 10 to 15 | | 5 |
| - 15 to 20 | | 0 |
| - 20 to 25 | | 5 |
| - 25 to 30 | | 0 |
| - 30 to 35 | | 2 |
| **Supplementary table S2: How many AOSD patients do you see / treat yourself annually (new patients and follow-up together)?** | | |
| **Average (90% CI)** | **5; 3,31-7,39** | |
| Average amongst academic hospitals (90% CI) | 7; 3,85-10,81 | |
| Average amongst general hospitals (90% CI) | 3; 2,12-4,13 | |
| - 0 to 5 | 9 | |
| - 5 to 10 | 6 | |
| - 10 to 15 | 0 | |
| - 15 to 20 | 1 | |
| - 20 to 25 | 1 | |

| **Supplementary table S3: Statements and results of Delphi survey** | | | |
| --- | --- | --- | --- |
| **Statement** | **Agreement** | **Level of evidence** | **Grade** |
| **Similarity to Still’s disease** |  |  |  |
| 1.1 The clinical presentation of Still's Disease in children is very similar to that of adults and therefore these conditions can be considered as expressions of the same disease spectrum. | 100% | 3B | C |
| 1.2 Early use of biologicals can improve outcomes in adults with AOSD | 100% | 5 | D |
| **Diagnosis** |  |  |  |
| 2.1 In case of suspicion of AOSD, we recommend assessing the Yamaguchi classification criteria to support diagnosis. | 94% | 5 | D |
| 2.2 Important differential diagnoses for AOSD are infections malignancies, systemic diseases, vasculitis and other auto-inflammatory syndromes. In case of suspicion, these diagnoses should be excluded on indication. | 100% | 5 | D |
| 2.3 Early diagnosis and treatment can prevent complications from AOSD. Therefore, a well-defined diagnostic work-up is essential in forms of AOSD that do not respond to NSAID monotherapy | 100% | 5 | D |
| 2.4 In addition to standard laboratory tests including blood cultures, serum ferritin should be determined and determination of soluble IL-2 receptor (sIL-2R) and possibly IL-18 should be considered as more sensitive inflammation markers, preferably before start glucocorticoids. | 100% | 5 | D |
| 2.5 With prolonged presence of fever or suspicion of an underlying lymphoma, a PET CT scan should be performed, if available in time, in accordance with the fever of unknown origin protocol, before starting treatment with glucocorticoids | 100% | 5 | D |
| 2.6 If MAS/HLH is suspected, a bone marrow puncture is performed, preferably before starting treatment with glucocorticoids | 100% | 5 | D |
| 2.7 In case of serious clinical features of suspected AOSD, completion of diagnostics should be aimed for within four days. | 100% | 5 | D |
| 2.8 In case treatment is started with a short-acting IL-1 blockade, diagnosis of alternative diagnoses is disrupted less than when starting glucocorticoids (after a possible decrease in serum for cytokines). | 100% | 4 | D |
| 2.9 In patients with suspected MAS and AOSD, MAS-targeted treatment should not be delayed even if all criteria are not met | 100% | 4 | C |
| **Treatment** |  |  |  |
| **Treatment with biological DMARDs** |  |  |  |
| 3.1.1 A trial treatment with short-acting IL-1 blockade can, in case of uncertainty about the diagnosis AOSD, contribute to the diagnosis | 88% | 5 | D |
| 3.1.2 If Il-1 blockade therapy is used as a diagnostic (so when in doubt about the diagnosis), the short-acting variant should be chosen primarily. | 100% | 5 | D |
| 3.1.3 If bacterial sepsis is unlikely, starting with short-acting IL-1 blockade in case of life-threatening and rapidly progressive AOSD is justified even if not all test results are known yet | 100% | 1B | B |
| 3.1.4 IL-1 blockade is an effective treatment early in the AOSD disease course, including in glucocorticoid naive patients | 88% | 2B | B |
| 3.2 In rapidly progressing AOSD, IL-1 blockade monotherapy without glucocorticoids can be started initially. If no clinical improvement occurs within 2 days, dose of short-acting IL-1 blockade can be doubled and or glucocorticoids added. | 82% | 5 | D |
| 3.3 IL-6 blockade is a treatment option in glucocorticoid-resistant or glucocorticoid-dependent AOSD | 100% | 2B | C |
| 3.4 IL-1 or IL-6 blockade is preferred in AOSD over TNF blockade, however TNF blockade may be useful in some patients with persistent arthritis (without systemic manifestations) in the course of their disease | 100% | 5 | D |
| 3.5 There is no evidence for difference in effectiveness between different forms of IL-1 blockade in the treatment of AOSD | 88% | 5 | D |
| 3.6 In case of life-threatening or rapidly progressing AOSD and preference of short-acting IL-1 blockade, start with 200 to 400 mg anakinra iv per day (off-label dose) in combination with glucocorticoids | 100% | 5 | D |
| **Treatment with glucocorticoids and MTX** |  |  |  |
| 4.1 If there is insufficient control of disease with mild AOSD NSAIDs, glucocorticoids may be added with or without MTX, whereby long-term high-dose glucocorticoids should be avoided. | 82% | 4 | C |
| 4.2.1 High dose glucocorticoids are effective in AOSD, but due to side effects, the aim should be to limit the frequency and duration of treatment | 100% | 2B | B |
| 4.2.2 In case of alarming symptoms, methylprednisolone iv pulse therapy is preferred over long-term high doses of oral preparations | 94% | 4 | D |
| 4.3 Adding MTX may be useful for the treatment of arthritis in AOSD, but there is little evidence for treatment in AOSD with systemic features. | 94% | 4 | C |
| 4.4 In case of life-threatening symptoms of AOSD, treatment with a combination of glucocorticoids with a biological or glucocorticoid monotherapy should be started as soon as possible. | 88% | 2B | C |
| **Disease remission/treatment tapering** |  |  |  |
| 5.1 Response to treatment in AOSD can be defined as decrease in serum ferritin, recovery of clinical symptoms, decrease in CRP and/or ESR | 100% | 5 | D |
| 5.2 Disease remission in AOSD can be defined as normalization of serum ferritin, absence of clinical symptoms, normalization of CRP and/or ESR | 94% | 5 | D |
| 5.3 In the treatment of AOSD, one should aim for clinical remission with no or low dose glucocorticoids (equivalent to prednisone ≤ 7.5 mg daily) | 88% | 5 | D |
| 5.4 If, after discontinuation of glucocorticoids, disease remission for 3 months has occurred while on IL-1 blockade tapering can be as follows:  - for short-acting IL-1 blockade 100mg s.c./d, taper by dosing every other day for 4-6 weeks until complete stop if remission persists.  - for long-acting IL-1 blockade therapy, tapering by increasing the interval of administration frequency: at dose 150mg s.c. extend interval by 1-2 weeks at a time. At interval 10-12 weeks and no symptoms of disease consider discontinuing administration). | 100% | 5 | D |

| **Supplementary table S4. Statements that did not reach consensus** | |
| --- | --- |
| **Statement** | **Agreement** |
| 1. In case of debilitating but not life-threatening complaints, such as arthritis, fever and rash, you should preferably wait for the diagnosis before starting treatment for AOSD | 76% |
| 1. In order to prevent glucocorticoid-induced side effects, disabling (but not life threatening) complaints such as arthritis, fever and rash can initially be treated with IL-1 blockade therapies without glucocorticoids. | 76% |
| 1. In the absence of clinical response to IL-1 blockade monotherapy within 1 week, increase the dose or add glucocorticoids. | 59% |
| 1. If fever still persists after 7 days, glucocorticoids should be added. | Not voted, decided to remove |

| **Supplementary table S5. Clinical evidence regarding use of glucocorticoids in AOSD** | | | |
| --- | --- | --- | --- |
| **Authors** | **Design** | **Number of patients** | **Reference** |
| Prendiville et al. | Case series | 5 | (11) |
| Ruscitti et al. | Multicentre cohort study | 80 | (12) |
| Cassidy | Review | Not applicable | (13) |

**Supplementary data S1. Search string for the use of glucocorticoids in AOSD**

“Adult onset Still’s disease” and “systemic GC”

Still’s disease and GC; restriction to clinical trials

**Supplementary data S2. Search string for the use of MTX in AOSD**

*Search 1*

Pubmed: AOSD+MTX: 158 results

Embase: AOSD+MTX: 613 results (excluding “conference abstracts”:511 results, including doubles from PubMed)

Final selection (screening title and abstract, selection on language (English) and randomized controlled trials, prospective and retrospective cohorts, and case series with significant number of participants: 12

Complete PubMed Search string:

AOSD (MeSH Term OR underlying terms in Title/Abstract) AND methotrexate (MeSH Term OR underlying terms in Title/Abstract)

((((adult onset still's disease[MeSH Terms]) OR (((Still's Disease, Adult-Onset[Title/Abstract]) OR (Stills Disease, Adult-Onset[Title/Abstract]) OR (Adult-Onset Still's Disease[Title/Abstract]) OR (Adult Onset Still's Disease[Title/Abstract]) OR (Adult-Onset Stills Disease[Title/Abstract]) OR (Still Disease, Adult Onset[Title/Abstract]) OR (Still Disease, Adult Onset[Title/Abstract]) OR (Adult-Onset Still Disease[Title/Abstract]) OR (Adult Onset Still Disease[Title/Abstract])))))) AND ((methotrexate[MeSH Terms]) OR (((methotrexate[Title/Abstract]) OR (Amethopterin[Title/Abstract]) OR (Methotrexate, (D)-Isomer[Title/Abstract]) OR (Methotrexate, (DL)-Isomer[Title/Abstract]) OR (Mexate[Title/Abstract]) OR (Methotrexate Sodium[Title/Abstract]) OR (Sodium, Methotrexate[Title/Abstract]) OR (Methotrexate, Sodium Salt[Title/Abstract]) OR (Methotrexate, Disodium Salt[Title/Abstract]) OR (Methotrexate Hydrate[Title/Abstract]) OR (Hydrate, Methotrexate[Title/Abstract]) OR (Methotrexate, Dicesium Salt[Title/Abstract]) OR (Dicesium Salt Methotrexate[Title/Abstract])))

Complete Embase Search string

AOSD (Explode/EMTREE Term OR underlying terms n in Title/Abstract/keyword) AND methotrexate (Explode/EMTREE Term OR underlying terms in Title/Abstract/keyword)

'adult onset still disease'/exp OR 'still/s disease, adult-onset':ti,ab,kw or 'still/s disease, adult onset':ti,ab,kw or 'adult-onset still/s disease':ti,ab,kw or 'adult onset still/s disease':ti,ab,kw or 'stills disease, adult-onset':ti,ab,kw or 'stills disease, adult onset':ti,ab,kw or 'adult-onset stills disease':ti,ab,kw or 'adult onset stills disease':ti,ab,kw or 'still disease, adult-onset':ti,ab,kw or 'still disease, adult onset':ti,ab,kw or 'adult-onset still disease':ti,ab,kw or 'adult onset still disease':ti,ab,kw AND '4 amino 10 methylfolic acid':ti,ab,kw OR '4 amino 10 methylpteroylglutamic acid':ti,ab,kw OR '4 amino n10 methylpteroylglutamic acid':ti,ab,kw OR 'a methopterine':ti,ab,kw OR 'abitrexate':ti,ab,kw OR 'amethopterin':ti,ab,kw OR 'amethopterine':ti,ab,kw OR 'ametopterine':ti,ab,kw OR 'antifolan':ti,ab,kw OR 'biotrexate':ti,ab,kw OR 'canceren':ti,ab,kw OR 'cl 14377':ti,ab,kw OR 'cl14377':ti,ab,kw OR 'emtexate':ti,ab,kw OR 'emthexat':ti,ab,kw OR 'emthexate’:ti,ab,kw OR ‘emtrexate’:ti,ab,kw OR ‘enthexate’:ti,ab,kw OR ‘farmitrexat’:ti,ab,kw OR ‘farmitrexate’:ti,ab,kw OR ‘farmotrex’:ti,ab,kw OR ‘folex’:ti,ab,kw OR ‘folex pfs’:ti,ab,kw OR ‘ifamet’:ti,ab,kw OR ‘imeth’:ti,ab,kw OR ‘intradose MTX’:ti,ab,kw OR ‘jylamvo’:ti,ab,kw OR ‘lantarel’:ti,ab,kw OR ‘ledertrexate’:ti,ab,kw OR ‘maxtrex’:ti,ab,kw OR ‘metex’:ti,ab,kw OR ‘methoblastin’:ti,ab,kw OR ‘methohexate’:ti,ab,kw OR ‘methotrate’:ti,ab,kw OR ‘methotrexat’:ti,ab,kw OR ‘methotrexat ebewe’:ti,ab,kw OR ‘methotrexate lpf’:ti,ab,kw OR ‘methotrexate preservative free’:ti,ab,kw OR ‘methotrexate sodium’:ti,ab,kw OR 3 ‘methotrexate sodium preservative free’:ti,ab,kw OR ‘methotrexato’:ti,ab,kw OR ‘methoxtrexate’:ti,ab,kw OR ‘methrotrexate’:ti,ab,kw OR ‘methylaminopterin’:ti,ab,kw OR ‘methylaminopterine’:ti,ab,kw OR ‘meticil’:ti,ab,kw OR ‘metoject’:ti,ab,kw OR ‘metothrexate’:ti,ab,kw OR ‘metothrexate sodium’:ti,ab,kw OR ‘metotrexat’:ti,ab,kw OR ‘metotrexate’:ti,ab,kw OR ‘metotrexin’:ti,ab,kw OR ‘metrex’:ti,ab,kw OR ‘mexate’:ti,ab,kw OR ‘mexate-aq’:ti,ab,kw OR ‘mexate-aq preserved’:ti,ab,kw OR ‘mpi 5004’:ti,ab,kw OR ‘mpi5004’:ti,ab,kw OR ‘MTX’:ti,ab,kw OR ‘neotrexate’:ti,ab,kw OR ‘nordimet’:ti,ab,kw OR ‘novatrex’:ti,ab,kw OR ‘nsc 740’:ti,ab,kw OR ‘nsc740’:ti,ab,kw OR ‘otrexup’:ti,ab,kw OR ‘rasuvo’:ti,ab,kw OR ‘reumatrex’:ti,ab,kw OR ‘rheumatrex’:ti,ab,kw OR ‘rheumatrex dose pack’:ti,ab,kw OR ‘sodium methotrexate’:ti,ab,kw OR ‘texate’:ti,ab,kw OR ‘texate-t’:ti,ab,kw OR ‘texorate’:ti,ab,kw OR ‘trexall’:ti,ab,kw OR ‘xaken’:ti,ab,kw OR ‘xatmep’:ti,ab,kw OR ‘zexate’:ti,ab,kw

**Supplementary data S3. Search string for the use of tocilizumab in AOSD**

*Search 1.*

Pubmed: AOSD+TCZ: 102 result

Embase: AOSD+TCZ: 295 results (excluding “conference abstracts”: 225 results, including doubles from PuMed)

Final selection (screening title and abstract, selection on language (English) and randomized controlled trials, prospective and retrospective cohorts, and case series with significant number of participants: 13

Complete PubMed search string

AOSD (MeSH Term OR underlying terms in Title/Abstract) AND tocilizumab (MeSH Term OR underlying terms in Title/Abstract)

((((adult onset still's disease[MeSH Terms]) OR (((Still's Disease, Adult-Onset[Title/Abstract]) OR (Stills Disease, Adult-Onset[Title/Abstract]) OR (Adult-Onset Still's Disease[Title/Abstract]) OR (Adult Onset Still's Disease[Title/Abstract]) OR (Adult-Onset Stills Disease[Title/Abstract]) OR (Still Disease, Adult Onset[Title/Abstract]) OR (Still Disease, Adult Onset[Title/Abstract]) OR (Adult-Onset Still Disease[Title/Abstract]) OR (Adult Onset Still Disease[Title/Abstract])))))) AND ((tocilizumab[MeSH Terms]) OR (((tocilizumab[Title/Abstract]) OR (RHPM-1[Title/Abstract]) OR (RG-1569[Title/Abstract]) OR (R-1569[Title/Abstract]) OR (MSB11456[Title/Abstract]) OR (MSB-11456[Title/Abstract]) OR (atlizumab[Title/Abstract]) OR (monoclonal antibody, MRA[Title/Abstract]) OR (RO 4877533[Title/Abstract]) OR (Actemra[Title/Abstract]) OR (roactemra[Title/Abstract]))))

Complete Embase search string

AOSD (Explode/EMTREE Term OR underlying terms n in Title/Abstract/keyword) AND tocilizumab (Explode/EMTREE Term OR underlying terms in Title/Abstract/keyword)

'adult onset still disease'/exp OR 'still/s disease, adult-onset':ti,ab,kw or 'still/s disease, adult onset':ti,ab,kw or 'adult-onset still/s disease':ti,ab,kw or 'adult onset still/s disease':ti,ab,kw or 'stills disease, adult-onset':ti,ab,kw or 'stills disease, adult onset':ti,ab,kw or 'adult-onset stills disease':ti,ab,kw or 'adult onset stills disease':ti,ab,kw or 'still disease, adult-onset':ti,ab,kw or 'still disease, adult onset':ti,ab,kw or 'adult-onset still disease':ti,ab,kw or 'adult onset still disease':ti,ab,kw AND 'tocilizumab'/exp OR 'actemra':ti,ab,kw OR 'actemra 200':ti,ab,kw OR 'atlizumab':ti,ab,kw OR 'lusinex':ti,ab,kw OR 'r 1569':ti,ab,kw OR 'r1569':ti,ab,kw OR 'roactemra':ti,ab,kw

**Supplementary Data S4**. **Search string for the use of TNF-blockade in AOSD**

Complete Pubmed search string

((((adult onset still's disease[MeSH Terms]) OR (((Still's Disease, Adult-Onset[Title/Abstract]) OR (Stills Disease, Adult-Onset[Title/Abstract]) OR (Adult-Onset Still's Disease[Title/Abstract]) OR (Adult Onset Still's Disease[Title/Abstract]) OR (Adult-Onset Stills Disease[Title/Abstract]) OR (Still Disease, Adult-Onset[Title/Abstract]) OR (Still Disease, Adult Onset[Title/Abstract]) OR (Adult-Onset Still Disease[Title/Abstract]) OR (Adult Onset Still Disease[Title/Abstract])))))) AND ((adalimumab[MeSH Terms]) OR (((adalimumab[Title/Abstract]) OR (infliximab[Title/Abstract]) OR (golimumab[Title/Abstract]) OR (etanercept[Title/Abstract]) OR (humira[Title/Abstract]) OR (remicade[Title/Abstract]) OR (simponi[Title/Abstract]) OR (monoclonal antibody, MRA[Title/Abstract]))))

Complete Embase search string

'still/s disease, adult-onset':ti,ab,kw or 'still/s disease, adult onset':ti,ab,kw or 'adult-onset still/s disease':ti,ab,kw or 'adult onset still/s disease':ti,ab,kw or 'stills disease, adult-onset':ti,ab,kw or 'stills disease, adult onset':ti,ab,kw or 'adult-onset stills disease':ti,ab,kw or 'adult onset stills disease':ti,ab,kw or 'still disease, adult-onset':ti,ab,kw or 'still disease, adult onset':ti,ab,kw or 'adult-onset still disease':ti,ab,kw or 'adult onset still disease':ti,ab,kw

AND

'infliximab'/exp OR 'remicade':ti,ab,kw

AND

'adalimumab'/exp OR 'humira':ti,ab,kw

AND

'etanercept'/exp OR 'enbrel':ti,ab,kw

AND

'golimumab'/exp OR 'simponi':ti,ab,kw

**Legends for supplementary figures**

Supplementary Figure S1: Expert-opinion-based Suggested treatment scheme for severe, non-life threatening AOSD with short-acting IL-1 blockade and GC**.** Double arrows indicate equivalent options and a decision should be made upon the physician’s discretion. Dose of GC added after insufficient stabilization 0,5-1,0 mg/kg body weight prednisone equivalent. GC= glucocorticoids. Short IL1, short-acting IL-1 blockade.

Supplementary figure S2: Expert opinion-based suggested treatment scheme for severe AOSD. Double arrows indicate equivalent options and a decision should be made upon the physician’s discretion.

Dose of GC in rapidly progressive disease: 0,5-1,0 mg/kg body weight prednisone equivalent. High GC in life threatening disease: 1000 mg prednisone equivalent during 3 days. IVIG= intravenous immunoglobulin (2g/kg every four weeks).
GC, glucocorticoid; Short IL1, anakinra.

Supplementary figure S3: Tapering scheme for treatment of AOSD as suggested by authors. Double arrows indicate equivalent options depending on what IL-1 inhibitor is used.

GC, glucocorticoid; IL1, IL-1 blockade; short IL1, anakinra; long IL1, canakinumab.
