# Supplementary figures and images for: Management of adult-onset Still's disease: evidence- and consensus-based recommendations by experts
Source: Rheumatology (Oxford). 2023 Sep 5;63(6):1656–63. doi: 10.1093/rheumatology/kead461 (PMC11147545; doi:10.1093/rheumatology/kead461)

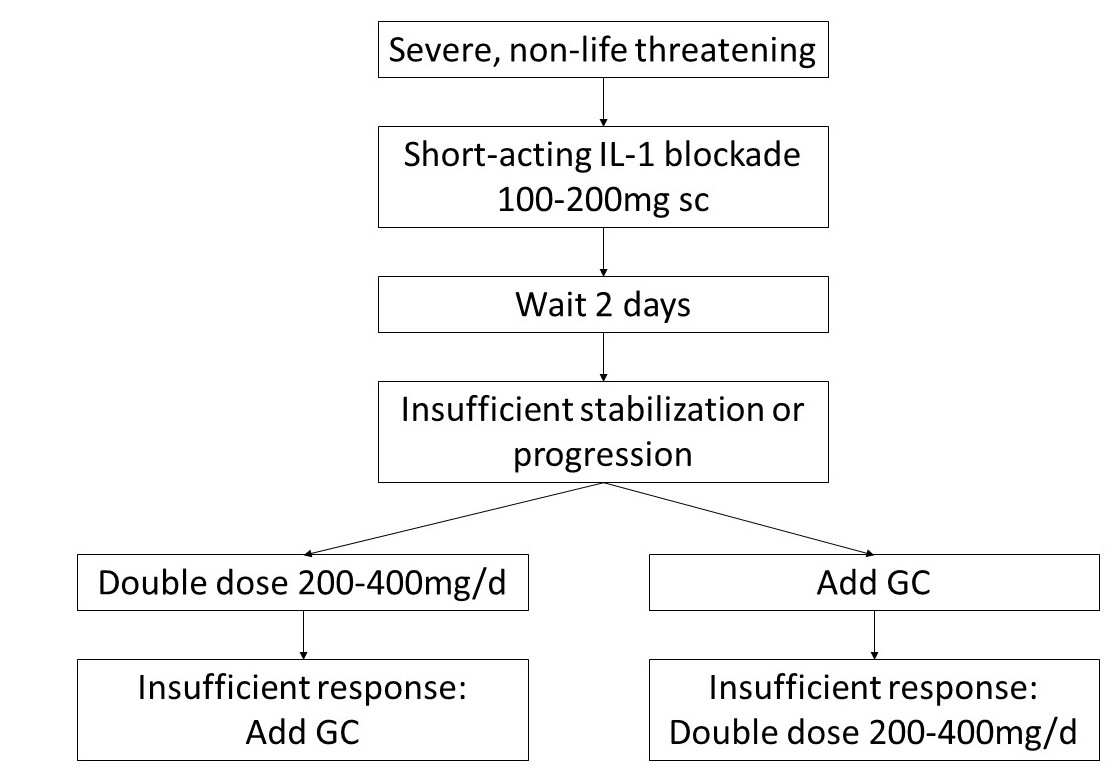

Supplement: kead461_Supplementary_Data [file kead461_supplementary_data.zip › kead461_Supplementary_Data/rhe-23-0590-File005.jpg]

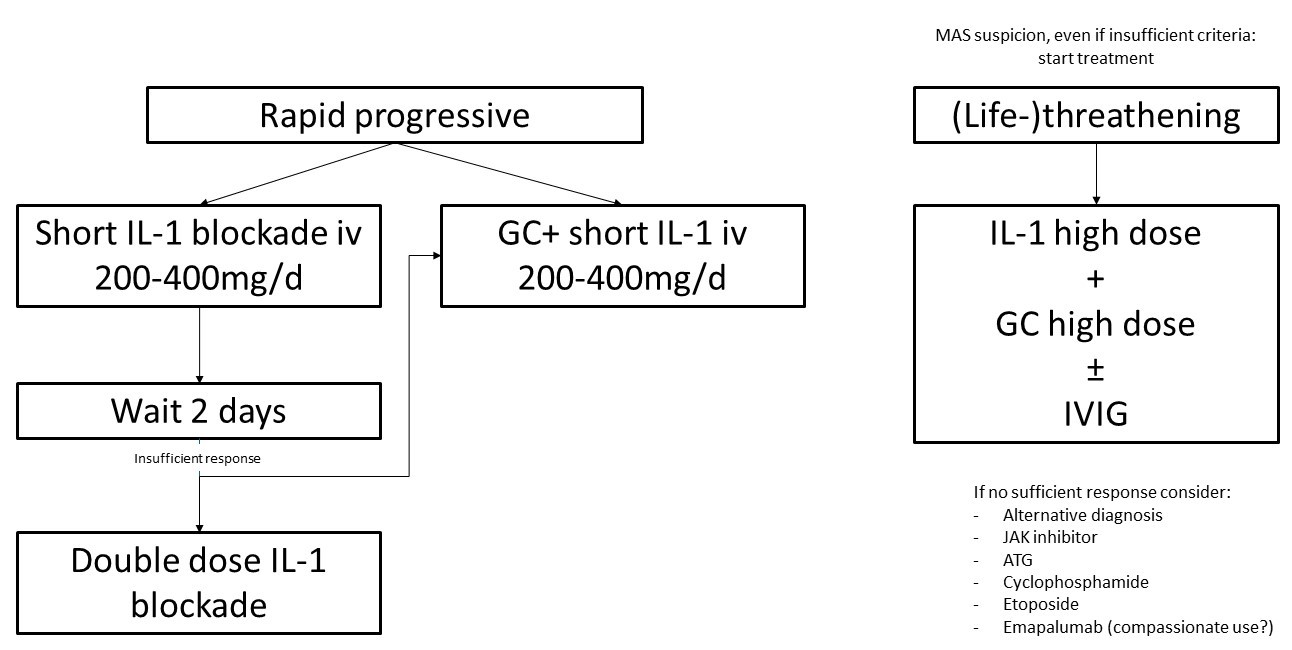

Supplement: kead461_Supplementary_Data [file kead461_supplementary_data.zip › kead461_Supplementary_Data/rhe-23-0590-File006.jpg]

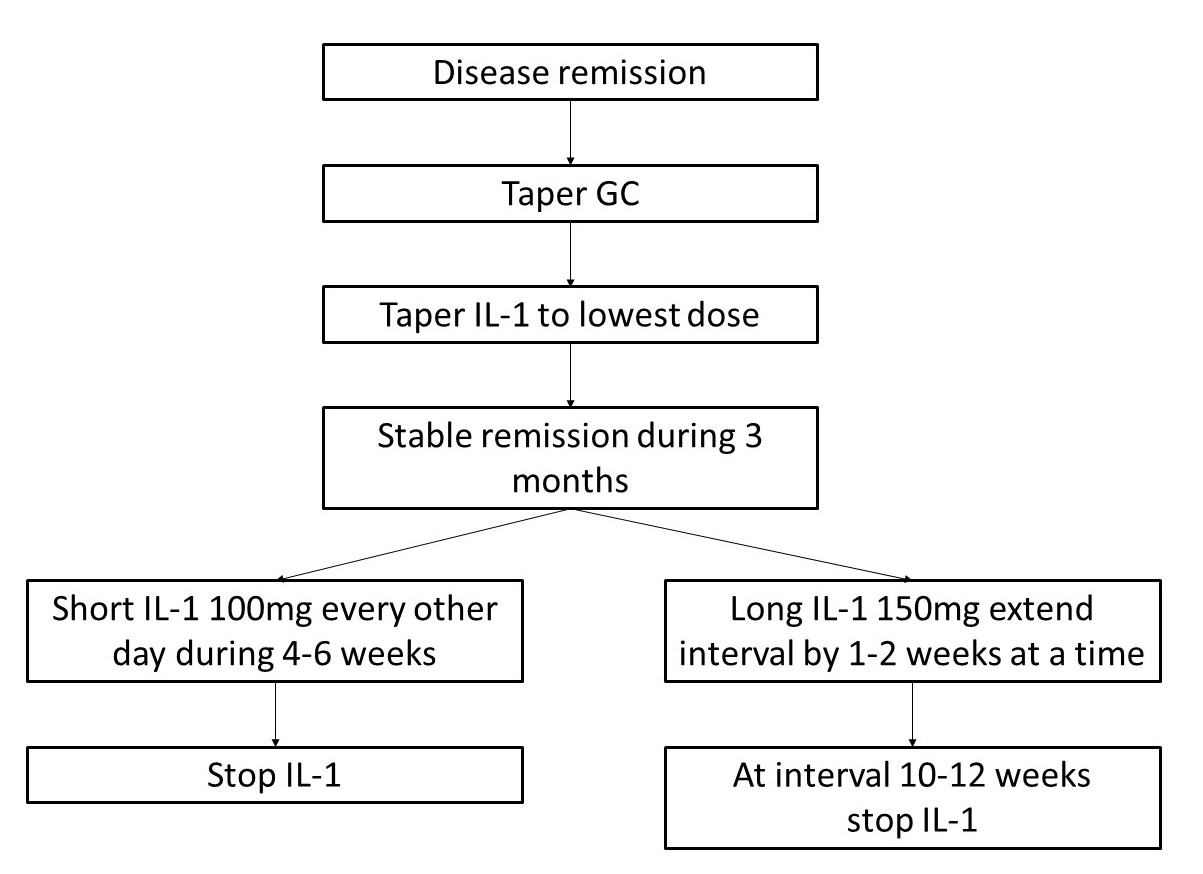

Supplement: kead461_Supplementary_Data [file kead461_supplementary_data.zip › kead461_Supplementary_Data/rhe-23-0590-File007.jpg]
